# Stem-cell-abundant proteins Nanog, Nucleostemin and Musashi1 are highly expressed in malignant cervical epithelial cells

**DOI:** 10.1186/1471-2407-8-108

**Published:** 2008-04-18

**Authors:** Feng Ye, Caiyun Zhou, Qi Cheng, Jiajie Shen, Huaizeng Chen

**Affiliations:** 1 Women's Reproductive Health Laboratory of Zhejiang Province, Women's Hospital, School of Medicine, Zhejiang University, Xueshi Rd #2, Hangzhou, 310006, China; 2 Department of Pathology, Women's Hospital, School of Medicine, Zhejiang University, Xueshi Rd #2, Hangzhou, 310006, China

## Abstract

**Background:**

Nanog, nucleostemin (NS) and musashi1 (Msi1) are proteins that are highly expressed in undifferentiated embryonic stem (ES) cells and have been shown to be essential in maintaining the pluripotency and regulating the proliferation and asymmetric division of ES cells and several nervous system tumor cells. The roles of Nanog, NS and Msi1 in development and progression of cervical carcinoma have, until now, not been well documented.

**Methods:**

In this study, expression of Nanog, NS and Msi1 was detected by immunohistochemistry analysis in 235 patients with various degrees of cervical epithelial lesions, including 49 with normal cervical epithelia, 31 with mild dysplasia (CIN I), 77 with moderate-severe dysplasia (CIN II-III) and 78 with squamous cervical carcinomas (SCCs). Associations with various clinical pathological prognostic variables were analyzed in 50 early-stage SCC patients.

**Results:**

Nanog, NS and Msi1 expression levels were significantly higher in SCC patients compared with CIN patients, and were higher in CIN patients compared with those with normal cervical epithelia. Nanog expression levels showed significantly differences according to different tumor sizes (P < 0.05), whereas there were no differences in NS and Msi1 expression levels according to different clinical pathological parameters.

**Conclusion:**

Our findings indicate that Nanog, NS and Msi1 may be involved in carcinogenesis of the cervix and progression of cervical carcinoma.

## Background

The stem-cell-abundant proteins Nanog, nucleostemin (NS) and Musashi1 (Msi1) are highly expressed in undifferentiated embryonic stem cells, and regulate stem-cell differentiation, proliferation and asymmetric division, respectively. Nanog is a unique homeobox transcription factor and has a homeodomain with homology to members of the natural killer (NK) gene family[1]; indeed, it has a similar critical role in regulating the cell fate of the pluripotent ICM (inner cell mass) during embryonic development, maintaining the pluripotent epiblast and preventing differentiation [2,3]. NS is a putative GTPase that binds to P53 and is highly expressed in the nucleoli of neuronal and embryonic stem cells, and several cancer cell lines. NS is essential for stem- and cancer-cell proliferation [4]. Msi1 is an RNA-binding protein that is abundantly expressed in neural stem/progenitor cells, astroglial progenitor cells and astrocytes in the vertebrate central nervous system[5] and regulates the expression of its target gene, mammalian numb (m-numb), at the translational level and is associated with asymmetric cell division in neural progenitor cells[6]. These three proteins may have roles in carcinogenesis of embryonic cancer (EC), gliomas, liver cancer, gastric cancer, and other cancers[7-9]. The roles of these proteins in the transformation of cervical epithelial cells and the occurrence and development of cervical carcinoma have not previously been investigated.

In this study, we examined the expression of Nanog, NS and Msi1 in cervical epithelial lesions of varying severity and in cervical carcinomas by immunohistochemical analysis and assessed their association with various prognostic variables.

## Methods

### Samples and Patients

The specimens (n = 235) were obtained from patients at the Women's Hospital, School of Medicine, Zhejiang University from October 2004 to June 2005. Of these 235 patients, 49 had normal cervical epithelia, 31 had mild dysplasia (CINI), 77 had moderate-severe dysplasia (CIN II-III) and 78 had squamous cervical carcinomas (SCC). None of the patients had recevied chemotherapy, immunotherapy, or radiotherapy prior to specimen collection. Of the 78 SCC patients, 50 were diagnosed with early-stage SCC (including 10 Ia, 38 Ib and 2 IIa patients) and underwent radical hysterectomy and pelvic lymphadenectomy. The other 28 patients were diagnosed with stage > IIa SCC.

This study was approved by the Medical Ethical Committee of Women's Hospital, School of Medicine, Zhejiang University. All patients signed informed consent to allow molecular research on specimen obtained during surgical operation.

### Primary Antibodies

The goat anti-human polyclonal antibodies specific for Nanog, NS and Msi1 were purchased from R&D (USA).

### Immunohistochemistry and Evaluation

Following sample collection, all tissues were immediately fixed in 10% neutralized formalin for 24 hours prior to transfer to paraffin wax using standard procedures. Paraffin sections (4 μm) were used for histological diagnosis or immunohistochemical analysis. Tissue sections were dewaxed and rehydrated using standard procedures. Hydrated autoclave pretreatment involved boiling for 2 minutes in 10 mM citrate buffer (pH 6.0). After cooling (20 minutes at room temperature), the sections were immersed in 3% hydrogen peroxide (H_2_O_2_) for 10 minutes to block endogenous peroxidase activity. Nonspecific staining was prevented by 10-minute incubation with normal rabbit serum (Maixin, China). Excess normal serum was removed and replaced with the primary antibody (Nanog antibody, 5 μg/ml; NS antibody, 5 μg/ml; Msi1 antibody, 10 μg/ml) and incubated for 2 hours in a humid chamber at room temperature. After washing, the sections were incubated with biotin-labeled anti-goat secondary antibody followed by avidin-biotin complex (ABC) for 30 minutes. These reagents were purchased from Maixin Corp. 3,3'-diaminobenzidine tetrahydrochloride (Dako, Germany) was added to visualize the reaction. Slides were washed three times (5 minutes each time) in phosphate-buffered saline (PBS)-Tween between each step. The slides were then counterstained with Mayer's hematoxylin, rinsed with tap water, dehydrated, placed in xylene, and mounted. Blank controls were performed by replacing primary antibodies with normal goat serum.

Positive cells were indicated by the presence of a distinct brown color in the nucleus or cytoplasm. The number of positively stained cells out of 100 in 10 random fields (400× objective) was counted and reported as the percentage of the total number of cells. The semiquantitative immunoreactive score ranged from - (0) to +++ (3) based on the percentage of positive cells and the stain intensity: - (0) = < 5% positive cells; +(1) = 5–25% positive cells; ++(2) = 26–75% positive cells; and +++(3) = more than 76% positive cells. Slides were independently reviewed by two pathologists and consensus agreements were reached.

### Statistics

The Kruskal-Wallis *H *test and the Mann-Whitney U test were performed using SPSS 13.0 software package for Windows. A level of 0.05 was chosen to indicate statistical significance. All reported *P *values were bilateral.

## Results

In CIN II-III and cervical carcinoma tissues, Nanog, NS and Msi1 were moderately or strongly expressed, in contrast to low expression levels in normal cervical epithelia and CIN I cells. All blank controls showed negative immunostaining results. The distinct brown color indicative of Nanog, NS and Msi1 expression was detected in the cytoplasm, nucleus and cytoplasm, respectively, of the positive cells.

**Figure 1:**
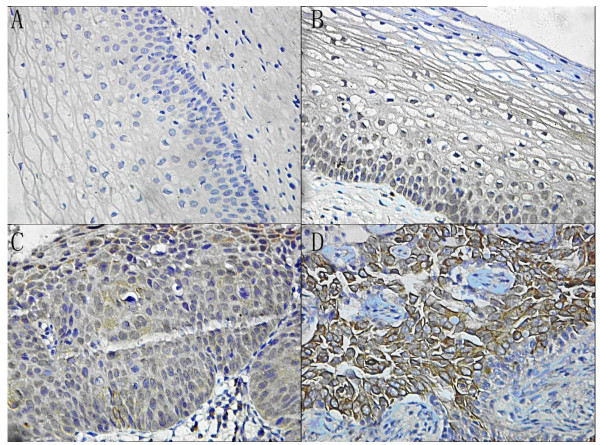
**Nanog expression and localization in (A) normal cervical epithelial cells; (B) CINI cells; (C) CINIII cell; (D) SCC cells**. The distinct brown coloration is located in the cytoplasm of the positive cells (400×).

**Figure 2:**
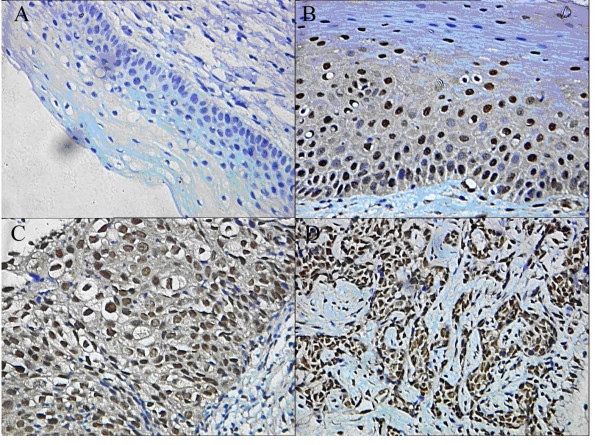
**Nucleostemin expression and localization in (A) normal cervical epithelial cells; (B) CINI cells; (C) CINIII cells; (D) SCC cells**. The distinct brown coloration is located in the nucleus or the cytoplasm of the positive cells (400×).

**Figure 3:**
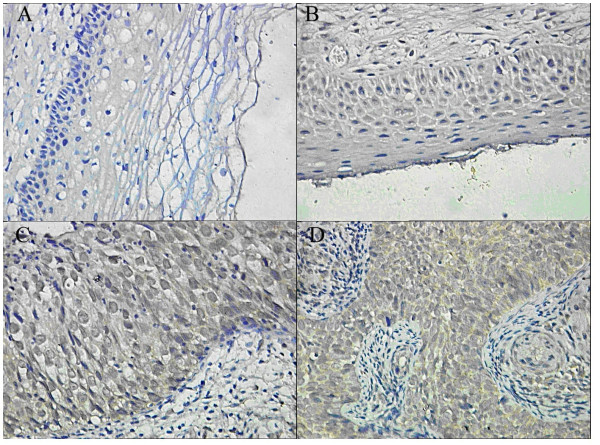
**Musashi1 expression and localization in (A) normal cervical epithelial cells; (B) CINI cells; (C) CINIII cells; (D) SCC cells**. The distinct brown coloration is located in the cytoplasm of the positive cells (400×).

Expression levels of Nanog, NS and Msi1 were higher in samples from SCC patients than in samples from patients with normal cervical epithelia and CIN; they were also higher in samples from patients with CIN than from those with normal cervical epithelia (Table 1). Typical staining of normal, CIN and SCC samples are shown in Figures 1, 2 and 3, respectively.

**Table 1:** Expression of Nanog, Nucleostemin and Musashi1 in cervical epithelial lesions of varying severity

		**Nanog**					**Nucleostemin**					**Musashi1**				
				
	**Total** N = 235	0(-) N = 26	1(+) N = 85	2(++) N = 76	3(+++) N = 48	**P**	0(-) N = 23	1(+) N = 70	2(++) N = 105	3(+++) N = 37	**P**	0(-) N = 26	1(+) N = 85	2(++) N = 76	3(+++) N = 48	**P**
Normal	49	17	22	8	2	**0.000**^†^	16	22	8	3	**0.000**^††^	15	25	7	2	**0.000**^†††^
CINI	31	2	18	9	2	0.009*	4	12	15	0	**0.017**^§^	3	13	14	1	**0.003**^¶^
CINII-III	77	4	25	35	13	0.015**	2	23	39	13	**0.008**^§§^	2	28	30	17	**0.039**^¶¶^
SCC	78	3	20	24	31	0.017***	1	13	43	21	**0.026**^§§§^	1	20	25	31	**0.032**^¶¶¶^

† P value of four groups, Chi-square = 49.720.

†† P value of four groups, Chi-square = 55.325.

††† P value of four groups, Chi-square = 51.013.

* Normal *vs *CINI, Z = -2.616;

** CINI *vs *CINII-III, Z = -2.429;

*** CINII-III *vs *SCC, Z = -2.397.

§Normal *vs *CINI, Z = -2.379;

§§ CINI *vs *CINII-III, Z = -2.669;

§§§CINII-III *vs *SCC, Z = -2.231.

¶Normal *vs *CINI, Z = -2.949;

¶¶ CINI *vs *CINII-III, Z = -2.064;

¶¶¶CINII-III *vs *SCC, Z = -2.146.

Associations between Nanog, NS and Msi1 expression levels and clinical pathological prognostic factors (such as age, clinical stage, tumor size, invasive depth, lymph nodes metastasis; lymph-vascular space invasion in parametrial tissues and differentiation, *et al*) were analyzed in samples from 50 early-stage SCC patients undergoing surgery. Only Nanog expression levels showed significant differences according to different tumor sizes (P = 0.049); NS and Msi1 expression levels showed no significant associations with the clinical pathological parameters analyzed (data not shown).

## Discussion

Nanog has an important role in regulating the cell fate of the pluripotent ICM during embryonic development, maintaining the pluripotent epiblast and preventing differentiation [2,3]. Nanog is also one of the key downstream effectors of several extrinsic signals that support the self-renewal and pluripotency of ES cells[10]. Increased levels of Nanog can maintain the mouse ES cell self-renewal ability independent of LIF (leukemia inhibitory factor) and allow human ES cell growth in the absence feeder cells[11]. Nanog is enriched in pluripotent cell lines such as ES, embryonic germ and EC cells, but it is not expressed in adult tissues; its expression is downregulated in differentiation tissues[12,13].

NS expression was closely associated with cellular proliferation in normal fibroblasts, T lymphocytes, bone marrow stem cells (BMCs), human placenta tissue, renal cell carcinoma cell lines and malignant renal tissues; but in terminally differentiated normal human adult kidney and mammary gland tissues, no NS expression could be detected [14,15]. It is likely to be a proliferation marker rather than a unique regulator of cell proliferation and survival in stem and cancer cells. In addition, Han *et al *reported that NS may have an important role in both tumorigenesis and the transformation of human embryonic bone marrow mesenchymal stem cells into F6 tumor cells because NS expression levels were markedly high in F6 cells [16]. Sijin *et al *showed that NS expression is required for HeLa cells to complete DNA synthesis and progress through S-phase: knockdown of NS expression led to increased numbers of HeLa cells in G0/G1 phase and the cell proliferation rate and *in vivo *tumorigenic capacity reduced markedly [17].

Msi1 is involved in maintenance of the character of progenitor cells, has an important role in regulating cell differentiation in precursor cells and is an evolutionarily well-conserved marker for neural stem cells and progenitor cells. Msi1 is an excellent marker for neural progenitor cells including neural stem cells in normal human brains [5]. Recently, Msi1 expression has been detected in human gliomas and melanomas, indicating it may be involved in oncogenic development. Tumors with high levels of Msi1 expression tended to have high levels of proliferative activity. Thus, the expression of Msi1 seems to be correlated with the grade of the malignancy and proliferative activity of gliomas, Msi1 may be a useful marker for the diagnosis of central nervous system tumors [7,18]. Shu *et al *detected the Msi1 protein in several human hepatoma cell lines, indicating that Msi1 expression may be an important factor in the development of several types of carcinoma and may be a useful molecular marker for tumor detection and diagnosis[8].

In our study, the distinct brown coloration indicative of Nanog, NS and Msi1 were located in the cytoplasm, nucleus and the cytoplasm, respectively, of the positive cells. Localization os NS and Msi1 were in accordance with previous reports; however, some reports have indicated that Nanog is localized to the nucleus. In the present study, we repeated the experiment and excluded the possibility of false-positive staining, and confirmed that Nanog protein was consistently localized to the cytoplasm. The mechanism by which localization occurs is unknown and further study is required.

There is an association among Nanog, NS and Msi1 expression levels and the severity of epithelial cell changes, with expression levels highest in cells from SCC, CIN and then normal cervical epithelium. However, there were no positive correlations among Nanog, NS and Msi1 expression levels and the clinical pathological prognostic factors analyzed, indicating that overexpression of these three stem-cell-abundant proteins in cervical epithlium is not related to the prognosis of cervical carcinoma[19].

Taken together, our results indicate that these three stem-cell-abundant proteins (Nanog, NS and Msi1 have roles in the carcinogenesis of cervical epithelial cells and regulate the cell differentiation, proliferation and asymmetric division, and maintain cancer cell pluripotency. Each protein may have an important and unique role in each step of the transformation process from normal cervical epithelial cells to malignant cells. Determining the molecules involved in tumorigenesis and development of cervical carcinoma is important for the study of this disease. Inhibition of the molecules important in the procession of tumor cell transformation may block the tumorigenesis of cervical carcinoma, and could have a clinical role in cervical carcinoma treatment; however, further study is needed.

## Competing interests

The author(s) declare that they have no competeing interests.

## Authors' contributions

FY collected the data, performed the statistical analysis and drafted the manuscript; CZ, QC and JS performed the immunoassays and pathologic examinations; HC designed the study concept, performed the statistical analysis, interpreted the results and approved the final manuscript. All authors read and approved the final manuscript.

## Pre-publication history

The pre-publication history for this paper can be accessed here:


